# Professional Development for Enhancing Autism Spectrum Disorder Awareness in Preschool Professionals

**DOI:** 10.1007/s10803-020-04562-9

**Published:** 2020-06-12

**Authors:** Linda Petersson Bloom

**Affiliations:** School Development and Leadership, Faculty of Education and Society, The National Agency for Special Needs Education and School (SPSM), Malmö University, Nordenskiöldsgatan 1, 211 19 Malmö, Sweden

**Keywords:** Autism spectrum disorder, Inclusive education, Preschool, Professional development

## Abstract

The current study describes the design, implementation, and analysis of a professional development programme using a Lesson Study model to enhance awareness in preschool professionals regarding inclusive education for children with autism spectrum disorder. The mixed method approach included pre- and post-intervention questionnaires, audio-recordings of group seminars, and an interview. The results indicated an increase in autism awareness among the professionals, suggesting that professionals changed their practice as a result of the programme. This was particularly clear regarding making adjustments to the learning environment and taking measures to prevent challenging situations. In addition to describing the implementation of a professional development programme in a preschool, this paper emphasises the importance of appropriate conditions for such initiatives.

## Introduction

The present study described and analysed the design and implementation of a professional development programme to enhance preschool in-service professionals’ awareness regarding children with autism spectrum disorder (ASD) and inclusive education. Professional development is often considered to be the main method for teachers to improve their professional skills (Cohen & Hill 2000). In the current professional development initiative, a Lesson Study (LS) model was used. The LS model is one of several collaborative professional development models with various designs (Holmqvist 2017). LS is typically used to enhance teachers’ capacity to develop students’ learning by instruction, and the focus is mainly on students’ learning during the intervention. In the current study, however, the focus was directly on teachers’ own learning about their awareness regarding ASD and inclusive education. The LS and iterative cycle design were based on the work of Marton and Tsui (2004), Holmqvist et al. (2007) and Langley et al. (2009). In the model, a problem is identified as something that needs to be improved, and changes are then developed and tested in practice.

**Fig. 1:**
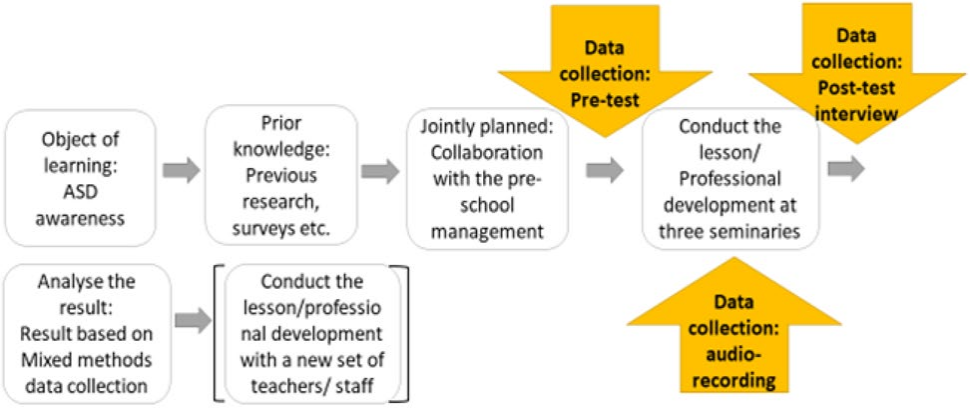
LS model for professional development used in the present study

As shown in Fig. 1, we conducted one complete LS cycle in the present study.

## Context of Swedish Early Education—Preschool

Although early education is not mandatory in Sweden, approximately 500,000 children aged 1–5 years old attend preschool (Swedish National Agency for Education [SNAE] 2019). Preschool education, which is included in the Swedish Education Act (Education Act 2010:800: SNAE), is coordinated by the Ministry of Education and Science. Preschool education represents the first step in the Swedish educational system (Lundqvist et al. 2016). The preschool curriculum has two parts: *The fundamental values and tasks of the preschool* and *Goals and guidelines*. Almost all children in Sweden with special educational needs are placed in regular preschools. Regarding this practice, the curriculum (Lpfö, 2018 SNAE) states, “Children who need more support and stimulation, either temporarily or permanently, should be provided with this, structured according to their own needs and conditions” (p. 7). This implies that preschool professionals must be able to meet the needs of these children and adjust the learning and teaching context to ensure that it is appropriate for all of the children at the preschool, including those with ASD. ASD is a group of neurodevelopmental disorders characterized by deficits in two areas: social communication and limited interest or repetitive behaviours (DSM-5; American Psychiatric Association, 2013). In a cohort study conducted in Stockholm that included children and youth with autism aged 0–27 years, the prevalence of ASD in children aged 0–5 years was estimated to be 0.40% (Idring et al. 2015). Although this prevalence was only measured in Stockholm, it may reflect the prevalence in other counties in Sweden.

Professionals in Swedish preschools include preschool teachers, who are educated at a university level, and childcare workers. Preschool teachers are responsible for planning education and evaluating the way in which the preschool offers opportunities for children to learn in accord with the goals of the curriculum (Lpfö 2018, SNAE). A recent study of preschool teachers in Sweden emphasized that structural factors, such as how many children are placed in each group and the number of professionals working at the preschool, can have consequences in terms of the quality of education (Persson & Tallberg Broman 2018). These factors were found to have particularly strong effects for younger children and children in need of special support and teaching. Furthermore, the authors noted that the entry requirements for preschool professionals have declined in recent years, reporting that preschool teachers are a high-risk group for burnout (Persson & Tallberg Broman 2018). The SNAE reported that there is currently a lack of preschool teachers in Sweden, and that only approximately 40% of professionals in preschools are preschool teachers. This represents a decrease from previous years (SNAE 2019).

## Professional Development Regarding ASD

Several previous studies have highlighted the importance of teachers and school professionals obtaining professional development regarding ASD (Alexander et al. 2015; Barnhill et al. 2014). School professionals must have a broad understanding of ASD and the needs of pupils with ASD in educational settings (Hart & Malian 2013). They also emphasized the importance of professional development for ASD awareness.

Moreover, some previous studies have observed that professionals who teach pupils with ASD are at an increased risk of burnout (Boujut et al. 2016; Coman et al. 2013; Ruble et al. 2013). However, there is some evidence that professional development in regard to ASD can be a protective factor for preventing teacher burnout (Coman et al. 2013; Corona et al. 2017; Jennett et al. 2003).

In addition, there are indications that teacher-efficacy can be a protective factor for preventing teacher burnout (Corona et al. 2017; Lauermann & König 2016; Ruble et al. 2011). Self-efficacy is defined as the degree to which a person feels that they are capable of meeting the challenges of the context in which they are operating. If a person thinks that they are sufficiently competent to handle a challenging situation, they tend to exhibit better performance (Bandura 1977). Some previous studies have investigated the relationship between self-efficacy in preschool teachers and their attitudes regarding inclusive education. Although one of these studies reported that there was such a relationship (Lifschitz & Glaubman 2002), two others did not find this connection (Sari et al. 2009; Engstrand & Roll-Pettersson 2014).

Importantly, people have been found to show more negative attitudes towards ASD compared with other disabilities (Huskin et al. 2018). Likewise, teachers have been found to have more positive perceptions of inclusive education in regards to students with physical or mild learning disabilities, compared with students with other disabilities, such as emotional disorders, cognitive impairment, or severe behavioural issues (Avramidis & Norwich 2002). However, some previous research has suggested that, compared with teachers of older students, elementary teachers and preschool teachers have more positive attitudes towards children with ASD, and inclusive education (Engstrand & Roll Pettersson 2014; Park & Chitiyo 2011).

All preschool professionals will likely be required to educate a child with ASD at some point during their career. Thus, interventions that enhance ASD understanding and awareness, as well as enabling professionals to create more inclusive educational settings for these children, should be prioritised. The current paper proposes that enhancing professional awareness of ASD could have a two-fold gain. First, children will receive enhanced education in better-equipped preschools, and, second, professionals will have greater feelings of teacher-efficacy, reducing the risk of burnout.

## Aim and Research Questions

The aim of the present study was to describe and analyse the design and implementation of a professional development programme on preschool professionals’ awareness of ASD. Furthermore, the aim is also to provide the professionals with new skills and strategies for improving the learning context, and thereby creating a more inclusive preschool, for children with ASD. In addition, the aim is to explore the process of how the preschool professionals change their practice during the intervention. Because the study included preschool teachers and child care workers, the term professionals was used when referring to both groups. The research questions were as follows:

RQ 1: What differences, if any, were found in attitudes regarding ASD awareness in preschool professionals after the intervention?
RQ 2: If changes were found, what examples of changed practice did the preschool professionals express? If changes were found, what examples of changed practice did the preschool professionals express?

In the current study, participant attitudes regarding ASD awareness included knowledge and understanding of ASD, as well as the comprehension and use of strategies to facilitate, adjust, and adapt the learning context. The present study relied on the notion that attitudes comprise cognitive, behavioural, and affective components (Boone & Kurtz 2002). Cognitive factors relate to beliefs, information, and knowledge, while affective factors refer to an individual’s feelings or reactions, and behavioural factors include actions made by an individual (Park et al. 2010). According to Lindblom et al. (2020), this framework can be translated to attitudes related to ASD and education. In present study, inspired by Lindblom et al. (2020), the cognitive component included knowledge about ASD. The affective component involved the way in which educational professionals perceive and react to children with ASD, and the behavioural component included the way in which educational professionals carry out their practice.

## Methods

### Design of the Intervention

**Fig. 2:**
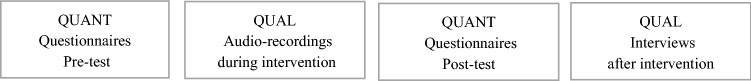
Study design

To answer the research questions, both quantitative and qualitative data were collected at different stages in the study process. The study had a mixed methods experimental design (Creswell & Creswell 2018). Pragmatism is the foundation of the mixed methods research approach, relying on the core assumption that multiple and diverse types of data provide a more complete understanding of a research question (Creswell 2007; Creswell & Creswell 2018). In the current study, qualitative data were collected during and after the intervention, as shown in Fig. 2.

The quantitative part of the experiment adopted a pre-experimental design, in which a single group was subjected to an intervention (Creswell & Creswell 2018).

### Participants and Setting

Cluster sampling was used in the sampling process in this study (Cohen et al. 2011). The sampling process started with the researcher directly contacting a team leader in one of the local authorities in the municipality (an urban city in the south of Sweden). The team leader worked in an area of the municipality that included several preschools. In the next step, the team leader informed principals in the area, and asked the preschool principals to contact the researcher. The principal at one preschool contacted the researcher and agreed to cooperate with the project.

Participants included in the study were preschool teachers and child care workers who worked at the preschool. The children at the preschool were placed in age-homogeneous groups. Children with ASD and other disabilities were also attending the preschool and placed in the regular units of the preschool. The preschool management comprised a principal, two deputy principals, and two senior preschool teachers.

A total of 18 participants completed the pre-test. As the preschool principals attended the first seminar but did not complete the pre- and post-tests, their data were not included in the analysis. One of the deputy principals also attended the second seminar.

The results of the study are based on the data from nine participants who completed both the pre- and post-tests and took part in at least two of the seminars. Of these participants, five were preschool teachers and four were child care workers. The number of years spent working at preschools varied widely, such that some professionals had only been working in preschools for 1–5 years while others stated that they had been working at preschools for more than 21 years. Although the age range of the children was relatively broad, most of the participants worked with children 3 to 4 years of age. Regarding the experience of working with children with ASD, four of the participants reported that they had limited experience, four reported that they had moderate experience, and one reported that they had extensive experience. Eight of the participants were women and one was a man.

### Procedure for the Professional Development Intervention

The professional development intervention consisted of three seminars that used an iterative process, spread over a period of 6 weeks. During the seminars, participants watched video-recorded lectures, which were part of a web-based professional development programme initiated by a Swedish governmental agency, The National Agency for Special Needs Education and Schools. The agency’s web-based professional development programme has five themes: Perspectives of parents and students with neurodevelopmental disabilities (NDD)/ASD, Cognition and perception in NDD/ASD, Strategies for use in learning contexts, Preventing challenging situations, and Accessibility (author’s translation). In the present study, four of the video-recorded lectures were chosen for inclusion in the professional development programme. The seminar topics were: accessibility (Lesson 1) and preventing challenging situations (Lessons 2 and 3). The video-recorded lectures served as “background” for the collaborative group discussions.

### Procedure for Data Collection

The questionnaires were administered before and after the intervention (i.e. pre- and post-test). The pre-test questionnaire was handed out at the first seminar. The questionnaires consisted of three background questions with closed-ended answers, six questions with closed-ended answers (Likert-scale), and seven questions with open-ended answers in combination with closed two-word alternative answers. For example, the following is a question from the pre- and post-test: “*I have sufficient competence regarding children with ASD, I have sufficient skills to adjust and adapt the learning context”*. In addition, the following is one of the open-ended questions: *Which strategies to instruct children with ASD do you know about*?” The questions were the same in the pre- and post-test questionnaires, but an additional question was included in the post-test questionnaire: *“How many times did you participate in the professional development?”* To enhance validity, the pre-test was conducted as near to the intervention as possible and the same questions were used in the pre- and post-tests (Creswell & Creswell 2018). The post-test was administered 5 weeks after the last seminar. The questionnaire was piloted on five professionals with a background in preschool education. The questionnaires were carried out in collaboration with Leifler (in press), who administered a similar professional development programme. Furthermore, the questionnaires, as well as the design of the intervention, were tested with senior researchers with experience with both questionnaires and interventions.

Audio-recordings were conducted during the group discussions. Since the group discussions were part of the intervention, the data collection was unobtrusive, increasing the validity of the study (Creswell & Plano Clarke 2011). The group discussions involved pre-determined questions. The focus was to facilitate the participants in identifying areas where they, as a preschool community, needed to develop and change to meet the needs of children with ASD and create a more inclusive preschool environment.

An interview was conducted 8 weeks after the last session. Although a group interview with at least two participants had been planned, circumstances at the preschool made it possible to interview only one participant. The interview followed a guide and was audio-recorded.

## Results

The results were organized and presented according to the data collection instruments. The pre- and post-test questionnaire data were analysed as descriptive and inferential statistics. The inferential statistics were analysed via the Wilcoxon test. All statistical analyses were conducted using SPSS version 25. The open ended-questions, data from group discussions, and interview data were thematised and compared using a thematic analysis (Braun & Clarke 2006). Data from the group discussions and the interview were transcribed. Conclusions were then made by synthesising the merged results. Importantly, the changes referred to in the results section were self-reported.

### Analysis of Closed-Ended Questions

Six of the participants stated in the pre-test that they did not have sufficient competence regarding ASD (Table 1). In the post-test, none of the participants disagreed with the statement that they had sufficient competence regarding teaching children with ASD. Six of the participants reported that they “moderately” agreed with the statement.

**Table 1:** Descriptive statistics pre- and post-test

Measure (n = 9)	Pre-test	Post-test
	Mean/ SD	Mean/ SD
Sufficient competence, ASD	Mean: 1.44	Mean: 2.33
	SD: 0.73	SD: 0.50
Sufficient preparation time	Mean: 2.00	Mean: 1.89
	SD: 0.00	SD: 0.33
Sufficient competence in making adjustments to learning environment	Mean: 1.56	Mean: 3.44
	SD: 0.53	SD: 0.53
Sufficient competence in preventing challenging situations	Mean: 1.44	Mean: 3.22
	SD: 0.53	SD: 0.44
Need for professional development	Mean: 3.33	Mean: 2.89
	SD: 0.71	SD: 0.93

Preparation time referred to the time the participants used to plan and prepare the educational programme for the children. All of the participants stated that they moderately agreed with the statement that they had sufficient preparation time in the pre-test. In the post-test, eight of the participants moderately agreed that they had a sufficient amount of preparation time, while one participant strongly disagreed. The results indicated that the more the professionals felt they knew, the more they also felt a lack of preparation time to design the activities to enhance participation for children with ASD.

In the pre-test, four participants disagreed and five moderately agreed that they had sufficient competence in adjusting the learning environment. After the intervention, the post-test showed that five participants strongly agreed and four very strongly agreed that they had sufficient competence in adjusting the learning environment.

One area of focus for the professional development programme was the ability to prevent challenging situations. Participants’ perceptions of their own competence shifted between the pre- and post-test, with five participants disagreeing that they had sufficient competence in the pre-test, and seven participants strongly agreeing with the statement in the post-test.

Participants were also asked to rate the degree to which they felt that they needed professional development regarding ASD. In the pre-test, four participants very strongly agreed, four strongly agreed and one of the participants moderately agreed with the statement. In contrast, in the post-test, four of the participants moderately agreed with the statement while two strongly agreed and three very strongly agreed. Interestingly, some participants perceived that they had a higher need for professional development after the intervention. This might indicate that the more the professional learned, the more they realised that they needed to gain knowledge.

The Wilcoxon test was used to investigate significant pre- and post-test differences. The results indicated that the median post-test rankings were significantly higher than the median rankings in the pre-test for three areas: Sufficient ASD competence post-test, *Mdn* = 2.00, and pre-test, *Mdn* = 1.00, *Z* = 2.83, *p* = 0.005; sufficient competence in making adjustments to the learning environment post-test, *Mdn* = 3.00, and pre-test, *Mdn* = 2.00, *Z* = 2.89, *p* = 0.004; and sufficient competence in preventing challenging situations post-test, *Mdn* = 3.00, and pre-test, *Mdn* = 1.00, *Z* = 2.81, *p* = 0.005. However, non-significant differences were found in the other two areas. Interestingly, there appeared to be an increase in the need for more preparation time and the need for professional development.

### Analysis of Open-ended Questions

When completing the open-ended questions, participants were asked to give examples of various factors, such as difficulties encountered by children with ASD when attempting to take part in a social task and different types of adjustments that participants had made to accommodate the students. Examples of participants’ responses are shown in Tables 2–4. The asterisks (“*”) indicate the themes that also emerged in the post-test data. Overall, the participants gave more examples with increased detail in the post-tests. One of the open-ended questions captured data regarding the consequences of different demands in social contexts involving children with ASD. Participants were asked to describe the demands that social contexts can place on children with ASD (Table 2).

**Table 2:** Participants’ examples regarding understanding of ASD in relation to social contexts

Pre-test	Post-test
Sounds/noises* Unclear environment*	Lack of appropriate adjustments to material/environment
Number of people/other children*	Insufficient preparation of activities and environment
Child does not understand expectations*	Unrealistic demands

The post-test responses revealed a greater emphasis on preparing the learning environment/context and adjusting for individual children. Furthermore, placing unrealistic demands on children’s abilities also emerged as a factor in the post-test responses, indicating that the focus shifted from the shortcomings of the children to the learning environment and context. Furthermore, the open-ended questions captured *Adjustments to the learning environment*. The first section included questions regarding adjustments that can be made to create a learning environment and context that is more predictable for children with ASD (Table 3).

**Table 3:** Participants’ examples regarding adjustments

Pre-test	Post-test
Visual support*	Visual support with variations. Individually designed visual support
Sign-supported speech*	Planning and preparation amongst professionals
Verbal explanations	Altering methods of explanation, emphasis on concrete information
Organisation of groups	Introduce activities one-on-one
Organisation of material*	Clarifying the beginning and the end of the activity Structured physical environment

Visual support was evident in both pre- and post-test responses. There appeared to be more qualitative and reflective development by the time the participants completed the post-test questionnaire. The professionals were more focused on variation and the provision of individually-designed visual support. Furthermore, participants stated that the visual support needed to be mobile. The participants identified that, as professionals, they needed to change and develop their approaches in providing certain forms of support. Table 4 shows examples of how the professionals worked to *prevent challenging situations.*

**Table 4:** Participants’ examples regarding preventing difficult situations

Pre-test	Post-test
Children do not understand the context.*	Use the “iceberg” metaphor
Children can become physical/act out	First understand the situation, then adjust the situation/context
Behaviour means something else, such as “I don’t understand”.*	Alter explanations, use concrete communication, be calm
Stand my ground regarding demands	Evaluate visual support and adjustments
Adjust the situation/demands.* Visual support	

The post-test data indicated that participants found new tools for understanding and preventing challenging situations, such as the “iceberg” metaphor, which can be used to describe behaviours and situations based on an understanding that the reason for something lies underneath the surface. While visual support was a theme in the pre-test responses, a different theme emerged in the post-test: evaluation (i.e. whether the visual supports and adjustments were appropriate for the individual child). As described above, the results suggested a further shift towards increased focus on the learning environment, such as context and changes that could be made. The professionals appeared to show increasing awareness regarding the need to alter their approach when working with children with ASD.

### Analysis of Group Discussion

The data analysis was focused on changes in the learning environment (Table 5). The table shows the outcomes of the participants’ collaborative process.

**Table 5:** Main outcomes after analysis and thematisation

Outcome seminar 1	Outcome seminar 2	Outcome seminar 3
Visual support Preparation Knowledge/understanding	Tools for understanding Wellbeing and stress in education professionals Lack of prerequisite skills	Tools for understanding Situations in which to use the tools Shared ideas and thoughts Lack of prerequisite skills Tying together the subject matter covered in the seminars

During the seminars, participants reported that many of the identified changes were related to the use of visual support and the “iceberg” metaphor to understand situations. Participants demonstrated a deepened understanding of some issues, such as the importance of visual support and making the environment and context predictable for children with ASD as a way of preventing challenging situations. To address the notion that children with ASD often need unique types of support, variation and mixtures of different types of visual support were recognized as areas that needed to be developed at the preschool. This pattern was also evident in the open-ended questions.

This was thought to be a core issue for creating a more inclusive education environment for children with ASD (as well as other children). One participant reported the following:

…we have been talking about the things we have discussed, and it is interesting that we also have learned that we need to learn even more. ASD is very complex and there is such variation in the children and what they need, but I think that we all need to learn more and complete more professional development.

Participants recognized that, to be able to use and develop visual support for children with ASD, they needed to be more prepared. Planning activities and lessons beforehand could facilitate the inclusion of appropriate visual support.

In addition, participants developed different ways to use the “iceberg” metaphor for understanding children’s behaviour, at an organizational level, and in meetings with parents.

Another area identified was knowledge and understanding regarding the individual needs and conditions required by children with ASD. Participants reported that it was important for them to obtain information about the needs of each child with ASD to provide appropriate support and adjustments.

In the process, participants also acknowledged that their own wellbeing and stress were important factors regarding their ability to cope with children in challenging situations.

A systematic lack of time for supporting children and for collaborative discussions was highlighted. In addition, participants emphasised the need for regular discussions between all of the professionals and members of the preschool management.

Moreover, participants identified that the changes they viewed as necessary were much harder to implement when only half of the professionals had taken part in the professional development programme. Hence, they identified a problem related to fidelity and implementation.

### Analysis of Interview Data

One participant who attended all three seminars was interviewed. The interview focused on changes that had been implemented after the professional development ended.

#### Changes in the Preschool

In the interview, the participant observed that there had been changes following the intervention at the preschool. The participant reported that these changes mostly related to the way that the education professionals understood, handled, and prevented different situations. For example, the participant made the following statement: “…Well, I think you could tell that we have changed our understanding, which is notable in the way we talk and discuss”. As shown in Table 4 and in the analysis of the group discussions, the “iceberg” metaphor appeared to have been adopted by the educational professionals. This was also identified in the interview, and the participant reported that some of the units at the preschool began to use the tool systematically. Furthermore, the ways in which professionals worked with visual support had also changed. More children were offered mobile visual support in a larger range of situations compared with before the intervention. The professionals also began to think earlier about how to conduct activities and what changes were necessary to make situations understandable and predictable for the children. The professional who was interviewed said: “Now we think earlier about what and how we need to change the learning environment and support, rather than just establishing that something isn’t working”.

Even though the focus in the professional development programme was ASD, some of the units had started to use visual support for children with other cognitive differences as well. Professionals made adjustments in the learning environment and context at an earlier stage for children with various needs. Although such changes had not necessarily been applied consistently across the entire preschool, a number of units had made changes and implemented new strategies.

#### Implementation

Amongst the participants, fidelity in terms of implementing the techniques learned during the intervention was relatively high, and the collaboration and discussions continued amongst the colleagues after the completion of the seminars.

One evident factor affecting implementation was that participants found it difficult to implement changes when less than half of the educational professionals at the preschool had attended the intervention. The professional who was interviewed stated: “Since we were so few participants it was very much up to us to try and take care of what we learned in the seminars, to implement and try to update our colleagues. It felt kind of lonely”.

In addition, the presence and participation of the school principals was mentioned as an important factor that was missing during the professional development programme. Finally, the participant expressed that they hoped the preschool would continue the professional development programme and extend it to include colleagues from other preschools in the area.

### Conclusions from the Synthesised Data

The quantitative analysis revealed statistically significant increases in three areas: Competence regarding ASD, competence in making adjustments to the learning environment and context, and competence in preventing challenging situations. The qualitative analyses supported the inferential analysis, indicating that there were several positive changes. When the results were merged, four main areas were aligned: *participant attitudes, visual support*, *tools for understanding*, *and implementation*.

#### Participant Attitudes

Participant attitudes regarding methods for creating an inclusive preschool setting appeared to change over the course of the programme. Prior to the intervention, participants had some notion that effective education for children with ASD should be approached from the perspective of optimising the learning environment and context to make it possible for the children to learn. The understanding and awareness of this concept appeared to increase during and after the professional development programme. This change was clearly described by the participant who was interviewed, and was also seen in the responses to the open-ended questions. There seemed to be a shift towards an increased focus on making changes to the learning environment or context to facilitate learning for children with ASD, which is an important factor. Furthermore, there seemed to be a shift towards an increased focus on the need for professionals to prepare effectively and plan activities in advance. Importantly, participants recognised their own wellbeing or lack of wellbeing as an important factor in their ability to cope with children in challenging situations.

#### Visual Support

The analysis indicated that the participants exhibited an evolving awareness of how to use visual support and how to apply it in various situations. Furthermore, participants seemed to exhibit an increased understanding of the way in which visual support design must be structured according to the needs and conditions of individual children. In conclusion, participants appeared to have obtained a broadened and deepened understanding and awareness of visual support as a result of the intervention.

#### Tools for Understanding

Taken together, the current data indicated that there is a need for preschool professionals to understand situations and behaviours in terms of ASD. The “iceberg” metaphor appeared to be a particularly enlightening tool for several of the participants, as indicated by the frequency with which it was discussed, and its repeated emergence as a theme in the group discussions. Participants also identified particular situations and gained awareness about what they could change and/or develop to make the preschool a more inclusive educational setting. Importantly, participants were easily able to identify situations that could be improved and possible approaches that could be used. However, because there is often not adequate time for planning and discussion in educational settings, these tools need to be simple, effective, and concrete to be useful to professionals.

#### Implementation and Prerequisites

Importantly, participants considered themselves, and the preschool as an organisation and community, to be lacking the prerequisite skills for working with children with ASD. This was revealed as a theme in the analysis of the group discussions, the interview, and the quantitative data. Concerning implementation, the data indicated that there were some issues that should be addressed. First, the participants stated that even though they found the intervention to be meaningful and useful, they found it difficult to implement it in the preschool as a whole. Furthermore, the social validity and fidelity of the intervention were relatively positive. This was revealed both in the analyses of the group discussions and the interview. Nonetheless, the high level of fidelity could also be explained by selection bias, if the participants who completed the professional development programme were more likely to have a positive view of the programme at the beginning.

The professional development programme appeared to meet the need for discussion among colleagues about developing their professionalism. Although the statistical analysis showed a trend towards a decrease in participants who rated their need for professional development as high, this was not statistically significant. This finding could suggest that the professional development programme was not sufficient. However, the results could also imply that the participants had gained more awareness and therefore were more aware that there is more to learn and understand. Furthermore, the importance of adequate organisational conditions for conducting professional development was qualitatively identified.

## Discussion

The goal of the current study was to investigate a professional development programme, designed as a LS cycle. The programme aimed to enhance awareness amongst educational professionals regarding methods for improving the learning context, and thereby changing their practice to create a more inclusive setting for children with ASD. Because this was a mixed methods study, interpretation of the results can span a broad range of considerations. The results revealed that attitudes regarding ASD awareness in preschool professionals changed after completing a professional development programme, and that they believed that the professional development programme had an influence on their practice. These results indicate that the professional development programme, designed as a LS, can serve as a starting point for enhancing ASD awareness and accommodation amongst preschool professionals.

### Implications for Practice

Although this was a small-scale study, the results have important implications. Previous reports have suggested that many teachers lack basic knowledge about ASD (Allday et al. 2013; Alexander et al. 2015; Barnhill et al. 2014). Although these previous studies examined teachers in school as opposed to preschool, the conclusions regarding personalized information and the lack of basic knowledge amongst teachers are likely to be useful for education professionals working with students with ASD of all ages.

Although the intervention in the present study had an impact on participant awareness and practice, regarding education for children with ASD, it is evident that educational professionals need professional development programmes that are more comprehensive. However, the present data indicate that even a small-scale collaborative professional development programme can induce some important changes in practice. The professionals expressed that they gained new skills and strategies, and reported that they felt they needed more competence after learning about the complexity of ASD and optimal learning contexts. This state can be described as uncertainty, which can be a precondition for learning (Munthe 2007).

It may be beneficial to think of inclusive education as an iterative process. This notion was emphasized by Humphrey (2008) in terms of willingness to develop and be flexible in a learning context. An iterative process and a willingness to make changes in the learning context were evident in the present study. Thus, the professionals were open to making changes and developing their practice, which is important for educational settings. Furthermore, adapting and adjusting the learning context are important factors in meeting the needs of children with ASD (Fleury 2014; Guldberg 2010; Humphrey 2008; Jordan 2005; Koenig et al. 2009).

The advantages of the professional development programme reported here suggest that it may be a beneficial tool for facilitating preschool professionals in fulfilling their responsibilities with respect to regulatory documents such as the Education Act and Curriculum.

### Study Limitations

The results of this study should be interpreted in consideration of several limitations. One important limitation is that the study population was relatively small. Thus, readers should be cautious when generalizing the findings of the study. Furthermore, the loss of participants may have affected the results. As mentioned in the Results section, the high fidelity with respect to the professional development programme could be explained by selection bias. However, the results may have been more positive if there had been more participants, leading to more professionals at the school making helpful changes and implementing new strategies learned during the programme. Although the qualitative data revealed changes in participants’ attitudes regarding ASD awareness and practice that were reflected, to some extent, in the quantitative data, the results cannot be extended to the whole preschool. Rather, it is possible to conclude that notable changes occurred in some units and in participants who attended at least two of the seminars. Moreover, it should be noted that the changes in attitudes regarding ASD awareness and practice were self-reported increases. Furthermore, the questionnaire used in this study had only been tested on five professionals, and had not previously been administered on a larger scale. Therefore, the validity and reliability of the test requires further examination. Importantly, there was no control group.

A more practical limitation is that the organization and structure of the professional development programme could have been more extensive and more clearly explained to preschool principals to increase participation. Interestingly, the dropout rate from the first to second seminar was relatively high. These dropouts were due to a number of staff members leaving the preschool, and the inability of some of the other professionals to attend for personal reasons.

## Conclusion

The current findings indicated that LS can act as a model for professional development, enabling professionals to work together to learn about ASD awareness and inclusive education. Furthermore, the iterative design appeared to contribute to a sense of ownership, because the model was focused on professionals identifying which changes should be made with respect to their practice. The results indicated that this small-scale programme provided participants with a deeper understanding of how to create an inclusive preschool setting for children with ASD. Furthermore, although the participants indicated that they did not feel that they had sufficient prerequisite skills to work with children with ASD, the programme exhibited a positive impact on their professional practice. Small actions can improve the inclusivity of the preschool environment for children with ASD.

## References

[CR1] Alexander JL, Ayres KM, Smith KA (2015). Training teachers in evidence-based practice for individuals with autism spectrum disorder: A review of the literature. Teacher Education & Special Education.

[CR2] Allday RA, Neilsen-Gatti S, Hudson TM (2013). Preparation for inclusion in teacher education pre-service curricula. Teacher Education & Special Education.

[CR3] American Psychiatric Association (2013). Diagnostic and statistical manual of mental disorders.

[CR4] Avramidis E, Norwich B (2002). Teachers’ attitudes towards integration / inclusion: A review of the literature. European Journal of Special Needs Education.

[CR6] Bandura A (1977). Self-efficacy: Toward a unifying theory of behavioral change. Psychological Review.

[CR7] Barnhill GP, Sumutka B, Polloway EA, Lee E (2014). Personnel preparation practices in ASD: A follow-up analysis of contemporary practices. Focus on Autism & Other Developmental Disabilities.

[CR8] Boone LE, Kurtz DL (2002). Contemporary marketing.

[CR9] Boujut E, Dean A, Grouselle A, Cappe E (2016). Comparative study of teachers in regular schools and teachers in specialized schools in France, working with students with an autism spectrum disorder: Stress, social support, coping strategies and burnout. Journal of Autism & Developmental Disorders.

[CR10] Braun V, Clarke V (2006). Using thematic analysis in psychology. Qualitative Research in Psychology.

[CR11] Cohen D, Hill H (2000). Instructional policy and classroom performance: the mathematics reform in California. Teachers College Records.

[CR12] Cohen L, Manion L, Morrison K (2011). Research methods in education.

[CR13] Coman D, Alessandri M, Novotny S, Gutierrez A, Boyd B, Hume K (2013). Commitment to classroom model philosophy and burnout symptoms among high fidelity teachers implementing preschool programs for children with autism spectrum disorders. Journal of Autism & Developmental Disorders.

[CR14] Corona LL, Christodulu KV, Rinaldi ML (2017). Investigation of school professionals’ self-efficacy for working with students with ASD: Impact of prior experience, knowledge, and training. Journal of Positive Behavior Interventions.

[CR15] Creswell JW (2007). Qualitative inquire and research design.

[CR16] Creswell JW, Plano Clark VL (2011). Designing and conducting mixed methods research.

[CR17] Creswell JW, Creswell DJ (2018). Research design, qualitative, quantitative, and mixed methods approaches.

[CR18] Education Act. 2010:800. Stockholm: Swedish Code of Statutes.

[CR19] Engstrand RZ, Roll-Pettersson L (2014). Inclusion of preschool children with autism in Sweden: Attitudes and perceived efficacy of preschool teachers. Journal of Research in Special Educational Needs.

[CR20] Fleury VP, Hedges S, Hume K, Browder DM, Thompson JL, Fallin K (2014). Addressing the academic needs of adolescents with autism spectrum disorder in secondary education. Remedial & Special Education.

[CR21] 10.1177/0741932513518823

[CR22] Garrad TA, Rayner C, Pedersen S (2019). Attitudes of Australian primary school teachers towards the inclusion of students with autism spectrum disorders. Journal of Research in Special Educational Needs.

[CR23] Guldberg K (2010). Educating children on the autism spectrum: Preconditions for inclusion and notions of “best autism practice” in the early years. British Journal of Special Education.

[CR24] Hart JE, Malian I (2013). A state wide survey of special education directors on teacher preparation and licentiate in autism spectrum disorders: A model for university and state collaboration. International Journal of Special Education.

[CR25] Holmqvist M (2017). Models for collaborative professional development for teachers in mathematics. International Journal for Lesson & Learning Studies.

[CR26] Holmqvist M, Gustavsson L, Wernberg A (2007). Generative learning: earning beyond the learning situation. Educational Action Research.

[CR27] Humphrey N (2008). Including pupils with autistic spectrum disorders in mainstream schools. Support for Learning.

[CR28] Huskin PR, Reiser-Robbins C, Kwon S (2018). Attitudes of undergraduate students toward persons with disabilities: Exploring effects of contact experience on social distance across ten disability types. Rehabilitation Counselling Bulletin.

[CR29] Idring S, Lundberg M, Sturm H, Dalman C, Gumpert C, Rai D (2015). Changes in prevalence of autism spectrum disorders in 2001–2011: Findings from the Stockholm youth cohort. Journal of Autism & Developmental Disorders.

[CR30] Jennett HK, Harris SL, Mesibov GB (2003). Commitment to philosophy, teacher efficacy, and burnout among teachers of children with autism. Journal of Autism & Developmental Disorders.

[CR31] Jordan R (2005). Managing autism and Asperger’s syndrome in current educational provision. Pediatric Rehabilitation.

[CR32] Koenig KP, Bleiweiss J, Brennan S, Cohen S, Siegel DE (2009). The ASD Nest program: A model for inclusive public education for students with autism spectrum disorders. TEACHING Exceptional Children.

[CR33] Langley GJ, Moen R, Nolan KM, Nolan TW, Norman CL, Provost LP (2009). The improvement guide: A practical approach to enhancing organizational performance.

[CR34] Lauermann F, König J (2016). Teachers’ professional competence and wellbeing: Understanding the links between general pedagogical knowledge, self-efficacy and burnout. Learning and Instruction.

[CR35] Lifshitz H, Glaubman R (2002). Religious and secular students’ sense of self-efficacy and attitudes towards inclusion of pupils with intellectual disability and other types of needs. Journal of Intellectual Disability Research.

[CR36] Lindblom A, Dindar K, Soan S, Karna E, Roos C, Carew MT (2020). Predictors and mediators of European student teacher attitudes toward autism spectrum disorder. Teaching & Teacher Education.

[CR37] Lundqvist J, Allodi Westling M, Siljehag E (2016). Characteristics of Swedish preschools that provide education and care to children with special educational needs. European Journal of Special Needs Education.

[CR38] Marton F, Tsui AB (2004). Classroom discourse and the space of learning.

[CR39] Muthe E, Zellermayer M, Munthe E (2007). Recognizing uncertainty and risk in the development of teachers’ learning communities. Teachers learning in communities: International perspectives.

[CR40] Park M, Chitiyo M, Choi YS (2010). Examining pre-service teachers’ attitudes towards children with autism in the USA. Journal of Research in Special Educational Needs.

[CR41] Park M, Chitiyo M (2011). An examination of teacher attitudes towards children with autism. Journal of Research in Special Educational Needs.

[CR42] Persson, S., & Tallberg Broman, I. (2018) *Nationellt uppdrag - lokala förutsättningar: Rapport 1 i projektet: Dilemma i förskollärares uppdrag: en studie mot bakgrund av ökad psykisk ohälsa bland förskollärare.* [National mission - local conditions: Report 1 of the project: Dilemma in the work of preschool teachers: a study on the background of increased mental illness among preschool teachers.] [In Swedish] Malmö University: https://muep.mau.se/handle/2043/24982

[CR43] Ruble LA, Usher EL, McGrew JH (2011). Preliminary investigation of the sources of self-efficacy among teachers of students with autism. Focus on Autism & Other Developmental Disabilities.

[CR44] Ruble LA, Toland MD, Birdwhistell JL, McGrew JH, Usher EL (2013). Preliminary study of the autism self-efficacy scale for teachers (ASSET). Research in Autism Spectrum Disorders.

[CR45] Sari H, Celikoz N, Secer Z (2009). An analysis of pre-school teachers’ and student teachers’ attitudes to inclusion and their self-efficacy. International Journal of Special Education.

[CR46] Swedish National Agency for Education (2019). Curriculum for the preschool Lpfö 2018.

[CR47] Swedish National Agency for Education. (2019). *Statistics on children and staff in preschool.* [In Swedish]. Skolverket. https://www.skolverket.se/skolutveckling/statistik/arkiverade-statistiknyheter/statistik/2019-04-09-statistik-om-barn-och-personal-i-forskolan

